# Mogroside V Alleviates Renal Injury in Diabetic Mice via Regulation of the *TLR4*/*NF-κB* Pathway and Modulation of ECM Remodeling

**DOI:** 10.3390/ijms27146271

**Published:** 2026-07-14

**Authors:** Xiangyu Guo, Hanzhe Shao, Jing Zhang, Xiangrong Xie, Dongcheng Peng, Qin Xu

**Affiliations:** Department of Pharmacy, Guilin Medical University, Guilin 541199, China

**Keywords:** mogroside V, *TLR4*/*MyD88*/*NF-κB* pathway, inflammation, ECM

## Abstract

Diabetic kidney disease (DKD) is a leading cause of end-stage renal disease, yet effective therapeutic strategies targeting its underlying mechanisms remain limited. Mogroside V (MV), a natural saponin from Siraitia grosvenorii, exhibits anti-inflammatory and antioxidant properties, but its role in DKD is unclear. This study investigated the effects and mechanisms of MV on renal injury in DKD using db/db mice and high-glucose-induced human renal mesangial cells (HRMCs). MV (25–100 mg/kg/d) was orally administered to db/db mice for eight weeks; HRMCs were treated with 0.5–2 μM MV under high-glucose conditions. Renal function, pathological changes, the expression of *TLR4*/*MyD88*/*NF-κB* pathway components, inflammatory cytokines, apoptosis-related factors, and ECM markers were assessed. MV significantly reduced fasting blood glucose, proteinuria, serum creatinine, and urea nitrogen levels, and ameliorated renal pathological injury in db/db mice. Mechanistically, MV downregulated *TLR4*, *MyD88*, and p-NF-κBp65 expression, suppressed inflammatory cytokine release (TNF-α, IL-1β, IL-6, MCP-1, IL-18), attenuated apoptosis (increased *Bcl-2*/*Bax* ratio, decreased *caspase-3*), and reduced ECM accumulation (decreased *MMP-9*, *Col IV*) both in vivo and in vitro. These findings suggest that MV protects against diabetic kidney disease, potentially by regulating the *TLR4*/*NF-κB* signaling pathway and improving extracellular matrix remodeling.

## 1. Introduction

Diabetic kidney disease (DKD) is one of the most common and severe microvascular complications of diabetes mellitus and a leading cause of end-stage renal disease (ESRD) worldwide [1,2]. Its pathological features include glomerular hypertrophy, thickening of the glomerular basement membrane, mesangial expansion, excessive accumulation of extracellular matrix (ECM), and tubulointerstitial fibrosis, ultimately resulting in progressive loss of renal function [3,4,5]. Although strict glycemic and blood pressure control can delay disease progression to some extent in clinical practice, the prevalence of DKD continues to rise, and current therapeutic strategies remain insufficient to halt its progression to ESRD [6,7,8]. Therefore, there is an urgent need to identify novel therapeutic targets and develop effective interventions to delay or reverse the progression of DKD.

The pathogenesis of DKD is complex and involves multiple interrelated mechanisms, among which chronic low-grade inflammation plays a central role [9,10]. Human renal mesangial cells (HRMCs) are key resident cells of the glomerulus and serve as critical effectors in DKD progression [11,12]. Under high-glucose conditions, HRMCs undergo abnormal proliferation, excessive accumulation of extracellular matrix, and the release of inflammatory cytokines [13,14,15]. The Toll-like receptor 4 (*TLR4*)/myeloid differentiation factor 88 (*MyD88*)/nuclear factor-kappa B (*NF-κB*) signaling pathway is a key driver of renal inflammation [16,17,18]. In the diabetic milieu, hyperglycemia and advanced glycation end-products activate *TLR4*, which recruits *MyD88* and triggers the phosphorylation and nuclear translocation of *NF-κB*, leading to the transcription of various pro-inflammatory cytokines such as tumor necrosis factor-α, interleukin-1β, and monocyte chemoattractant protein-1 [17,19,20]. Elevated expression of *TLR4* and its downstream effectors has been consistently observed in the kidney tissues of both DKD patients and animal models [21,22]. Furthermore, the inhibition of this pathway in experimental models has been shown to attenuate renal inflammation, reduce extracellular matrix deposition, and improve renal function [16,23,24].

**Figure 1:**
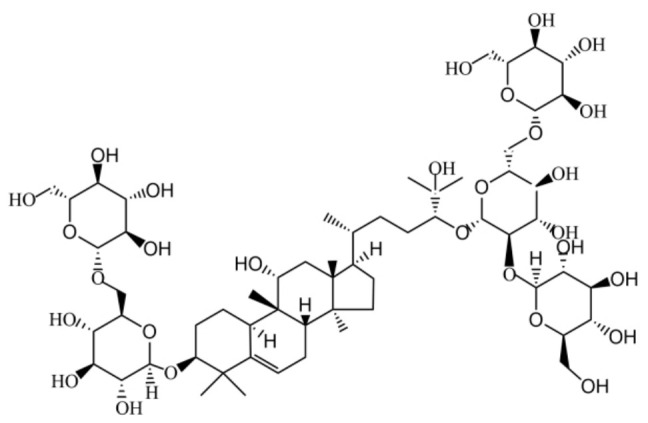
Chemical structural formula of Mogroside V.

Mogroside V (MV, Figure 1) is a tetracyclic triterpenoid saponin derived from *Siraitia grosvenorii*, a medicinal and edible plant, and possesses a variety of pharmacological activities, including anti-inflammatory, antioxidant, and hypoglycemic effects [25,26,27,28]. Notably, MV has been shown to suppress the *TLR4*/*MyD88*/*NF-κB* signaling cascade in models of pulmonary inflammation, thereby reducing the production of pro-inflammatory mediators [29,30,31]. Given that chronic inflammation driven by this signaling axis is a hallmark of DKD pathogenesis and that MV exhibits well-documented anti-inflammatory properties, it is plausible that MV may exert renoprotective effects in DKD through modulation of this pathway. Accordingly, this study aimed to investigate the protective effects of MV against DKD using db/db mice and high-glucose-induced HRMC models, with a focus on its regulation of the aforementioned signaling pathway. The findings are expected to provide a reference for the further development and utilization of MV and for the identification of quality markers of *Siraitia grosvenorii*.

## 2. Results

### 2.1. MV Can Improve the Clinical Symptoms of db/db Mice

The db/db mice were randomly allocated into groups at the outset, with no significant differences in weight. Following a four-week treatment period, the model db/db mice ceased to gain weight and experienced a significant reduction in weight from six to eight weeks (Table 1). Notably, after two weeks of oral administration of MV, there was no significant difference in weight between the treated db/db mice and their control counterparts. However, after four weeks of treatment with MV, the treated db/db mice showed marked weight gain, which was significantly different from that of model mice (*p* < 0.01). These findings suggest that MV can markedly reverse weight loss and effectively inhibit excessive weight loss in late-stage DKD-induced mice.

The blank group mice exhibited normal patterns of eating and drinking, sound mental state and vigilant reflexes, minimal excretion, and well-formed feces with sleek fur. By contrast, the model db/db mice presented obvious symptoms, such as poor mental state, significant weight loss, sluggish behavior, reduced activity time, dull and yellowed fur, excessive excretion, and watery feces. After eight weeks of continuous oral administration of both MV and Met, all treated mice experienced distinct improvements in these aforementioned symptoms.

**Figure 2:**
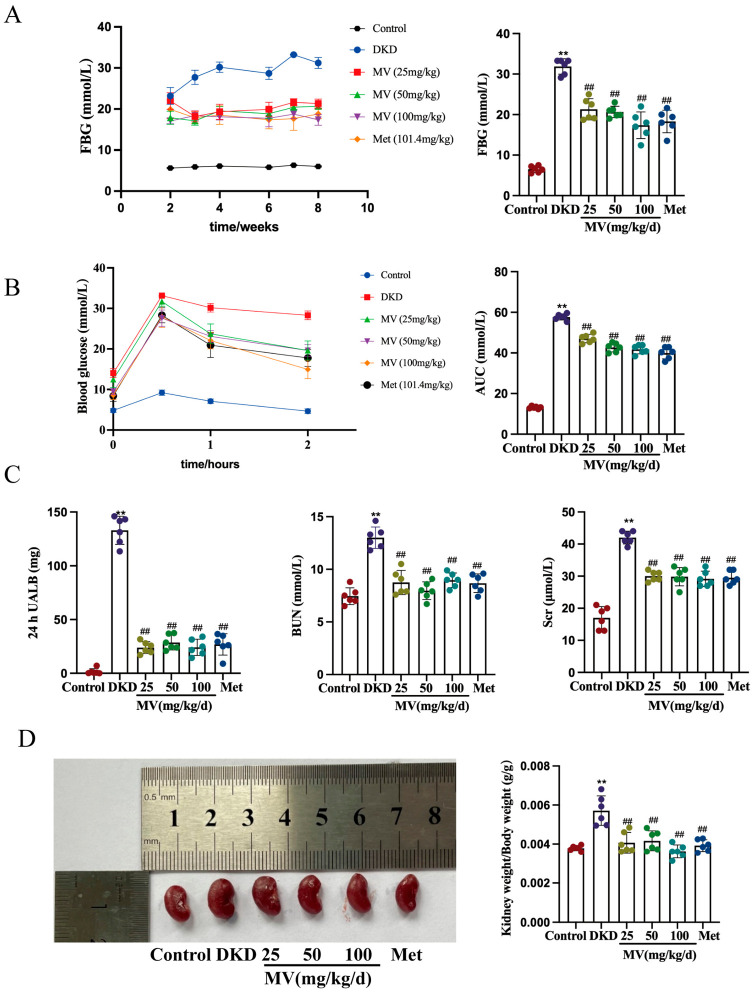
(**A**) Effect of MV on FBG in db/db mice (*n* = 6). (**B**) Effect of MV on OGTT in db/db mice (*n* = 6). (**C**) Effect of MV on 24 h UALB, BUN, Scr in db/db mice (*n* = 6). (**D**) Effect of MV on kidney in db/db mice (*n* = 6). ** *p* < 0.01 vs. control group, ## *p* < 0.01 vs. DKD group.

FBG levels were decided in each group of mice (Figure 2A). The blood glucose levels of treated db/db mice were significantly lower than those of model group mice. Specifically, among model db/db mice, their FBG levels remained high; however, after three weeks of oral administration of MV and Met, the treated db/db mice experienced a significant descend in FBG levels as compared to model group mice (*p* < 0.01). OGTT results (Figure 2B) revealed that MV significantly lowered the AUC in db/db mice (*p* < 0.01), indicating improved glucose tolerance.These findings infer that MV has a significant hypoglycemic effect and can markedly reduce FBG levels in db/db mice. We measured 24 h UALB, BUN, and Scr levels in each group of mice (Figure 2C). The 24 h UALB, BUN, and Scr levels of model mice were significantly higher than those of blank group mice (*p* < 0.01), indicating impaired kidney function in db/db mice. However, after eight weeks of MV and Met treatment, there was a decline in the levels of 24 h UALB, BUN, and Scr (*p* < 0.05, *p* < 0.01). These findings educe that MV can effectively reduce 24 h UALB, BUN, and Scr levels, alleviate symptoms of impaired kidney function in db/db mice, and have a protective effect on them.

The kidney index can reflect the renal damage in db/db mice. Model group db/db mice exhibited significantly enlarged kidneys and swelling (Figure 2D). After eight weeks of MV and Met treatment, the kidney volume and indices of db/db mice returned to normal levels, with significant reductions in kidney indices (*p* < 0.01). These findings suggest that MV can alleviate the degree of renal swelling in db/db mice, thereby improving renal tissue damage.

### 2.2. MV Promotes the Proliferation of HRMC Inhibited by HG

**Figure 3:**
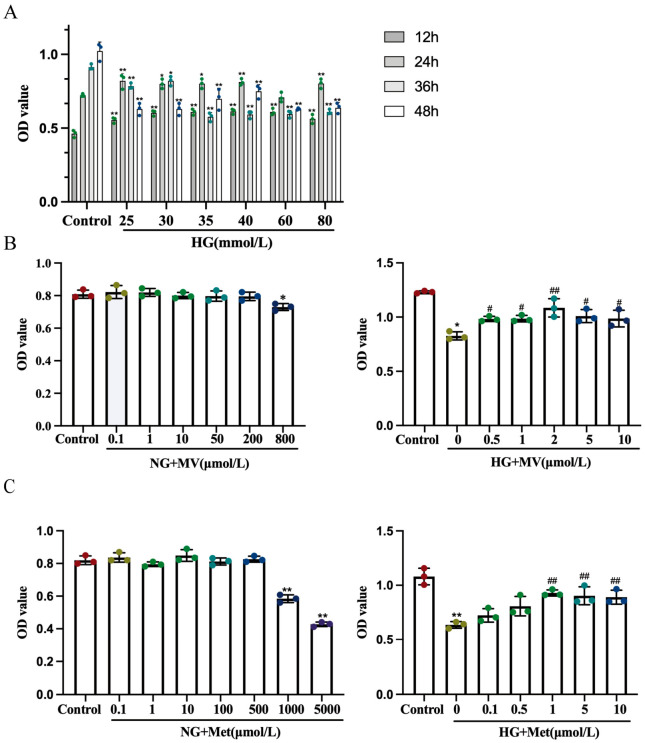
Screening of molding conditions, MV and Met concentrations. (**A**) The dose–effect and time–effect relationship of HRMC induced by HG ($\bar{x}$ ± s, *n* = 3) (**B**) Effect of MV on HRMC activity ($\bar{x}$ ± s, *n* = 3). (**C**) Effect of Met on HRMC activity ($\bar{x}$ ± s, *n* = 3). * *p* < 0.05 vs. respective control group, ** *p* < 0.01 vs. respective control group; # *p* < 0.05 vs. respective HG group; ## *p* < 0.01 vs. respective HG group.

When the concentration of HG reached 25 mmol/L and the action time spanned 48 h, the inhibitory effect on cell proliferation was the most obvious (*p* < 0.01), suggesting the injury of HRMC (Figure 3A). Therefore, 25 mmol/L HG was used to treat HRMC injury for 48 h as the model condition to induce HRMC injury. As shown in Figure 3B, MV of 0.5–10 μM could reverse the inhibitory effect of HG on HRMC and promote the proliferation of HRMC (*p* < 0.05, *p* < 0.01). Among them, 2 μM MV had the most obvious promoting effect on HRMC. Therefore, 0.5, 1 and 2 μM of MV were selected as low, medium and high therapeutic doses for follow-up experiments. Similarly, the administration of 0.5–10 μM Met could significantly increase the value-added rate of HRMC (*p* < 0.05, *p* < 0.01). Among them, the Met enhancement effect of 1 μM was the most obvious (Figure 3C). Therefore, 1 μM of Met was selected as the positive drug concentration.

### 2.3. MV Inhibited HG-Induced Apoptosis of HRMC

**Figure 4:**
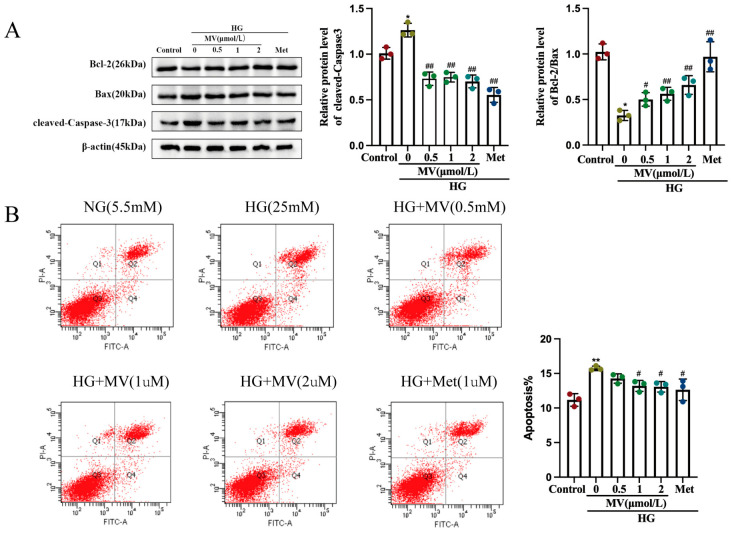
Effects of MV on HG-induced apoptosis of HRMC. (**A**) Western blot analysis was used to detect the expression of apoptosis-related protein *Caspase-3*, *Bax*, and *Bcl-2*. (**B**) The apoptosis rate of HRMCs was analyzed by flow cytometry with Annexin V-FITC (green fluorescence, x-axis) and PI (red fluorescence, y-axis) double staining; Q1 represents necrotic cells, Q2 late apoptotic cells, Q3 viable cells, and Q4 early apoptotic cells. * *p* < 0.05 vs. control group; ** *p* < 0.01; # *p* < 0.05 vs. HG group; ## *p* < 0.01 vs. HG group.

Western blot (Figure 4A) detected the expression of *Bcl-2*, *Bax* and cleaved *caspase-3* in HRMC to determine whether EPF had anti-apoptotic effect in HMC injury induced by HG. Compared to the control group, the expression of *caspase-3* protein and the mRNA of HRMC apoptosis-related factor in HG group were significantly increased (*p* < 0.01), while the protein and mRNA expression of *Bcl-2*/*Bax* was significantly decreased. After MV treatment, compared to HG group, the expression of *Caspase-3* protein decreased and the expression of *Bcl-2*/*Bax* protein increased. Met shows a similar trend. It is suggested that MV can increase the inhibitory factor of apoptosis, reduce the expression of pro-apoptotic factor and inhibit the apoptosis of renal cells.

In order to further determine whether MV can reduce the apoptosis of HRMC, flow cytometry was used to detect the apoptosis rate of HRMC, as shown in Figure 3B. Compared to control group, HG group was more prone to apoptosis (*p* < 0.05), while MV treatment group significantly reduced HG-induced apoptosis (*p* < 0.05).

### 2.4. Effect of MV on Pathological Injury of DKD

**Figure 5:**
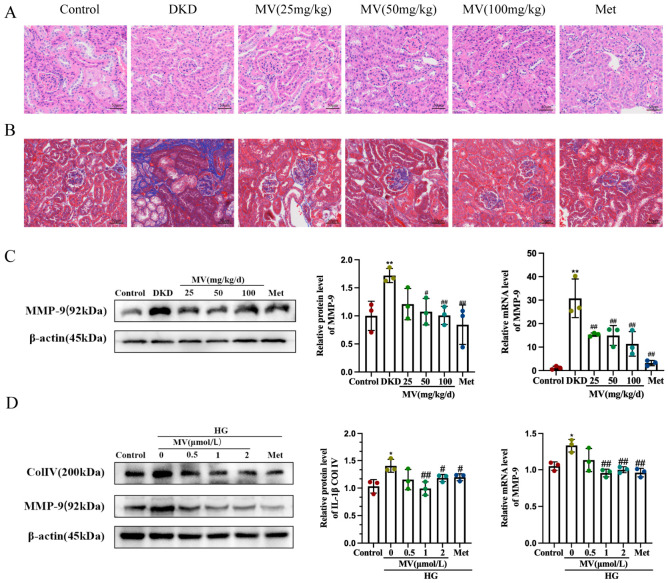
Effect of MV on pathological injury of DKD. (**A**) HE staining (400×). (**B**) Masson staining (400×). (**C**) Western blot and RT-qPCR analysis was used to detect the expression of *MMP-9* protein and mRNA in mouse kidney. (**D**) Western blot analysis was used to detect the expression of ECM-related protein *Col IV* and *MMP-9* in HRMC. * *p* < 0.05 vs. respective control group (normal mice for panel C, normal glucose HRMC for panel D); ** *p* < 0.01 vs. respective control group; # *p* < 0.05 vs. respective DKD group (DKD mice for panel C, HG-treated HRMC for panel D); ## *p* < 0.01 vs. respective DKD group.

In order to observe the pathological changes in the kidneys, we utilized the HE and Masson staining techniques to process the mouse renal tissues. The HE staining results (Figure 5A) showed no abnormalities in the glomeruli or tubules of the normal group mice’s kidneys, nor any significant thickening or hardening in the mesangial matrix. However, the model group db/db mice’s kidney sections displayed thickened glomerular basement membrane and increased mesangial thickness. After 8 weeks of treatment with MV or Met ingestion, the above conditions were alleviated. The Masson staining images (Figure 5B) indicated a significant increase in collagen deposition in the db/db mouse kidney model group, with fibrosis observed between the glomeruli and tubular interstitium. After 8 weeks of administration, the treated db/db mice showed significant improvements in their fibrotic condition, with a decrease in collagen deposition.

To further investigate the impact of MV on ECM, the expression of *MMP-9* protein and mRNA in mouse kidney was detected by Western blot and RT-qPCR, and the results showed that MV could inhibit it (Figure 5C). In addition, Western blot analysis was conducted to detect the protein expression of ColIV and *MMP-9* in HG-induced HRMC (Figure 5D). Compared to the control group, the protein levels of ColIV and *MMP-9* in the HG group HRMCs were significantly increased (*p* < 0.01). However, after treatment with MV or Met, the protein levels of ColIV and *MMP-9* were significantly reduced when compared to the HG group (*p* < 0.05, *p* < 0.01). This suggests that MV can alleviate the accumulation of extracellular matrix induced by HG.

### 2.5. Inhibitory Effect of MV on Inflammatory Response

**Figure 6:**
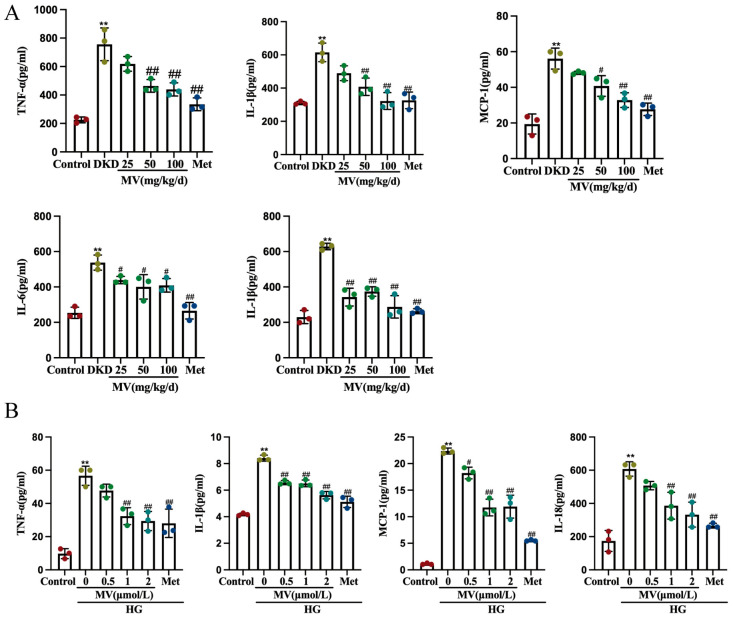
MV suppressed inflammatory responses. (**A**) The levels of IL-6, IL-18, IL-1β, TNF-α and MCP-1 in the supernatant were detected using ELISA after treatment with MV (25, 50, 100 mg/kg/d) or Met (101.4 mg/kg/d) in the serum of db/db mice (n = 3). (**B**) The levels of TNF-α, IL-1β, MCP-1 and IL-18 in the supernatant were detected using ELISA after treatment with HG in the presence or absence of MV or Met for 48 h in HRMC. ** *p* < 0.01 vs. respective control group; # *p* < 0.05 vs. respective DKD group (DKD mice for panel A, HG-treated HRMC for panel B); ## *p* < 0.01 vs. respective DKD group.

We utilized the ELISA method to detect the levels of MCP-1, TNF-α, IL-1β, IL-6, and IL-18 in the serum of different groups of db/db mice as well as in the supernatant of HG-induced HRMC. The result is shown in Figure 6A,B. The model group exhibited a significantly increase in the concentrations of TNF-α, IL-1β, MCP-1, IL-6, and IL-18 relative to the control group (*p* < 0.01). However, following treatment with MV and Met, the expression levels of these factors were significantly reduced (*p* < 0.05, *p* < 0.01).

**Figure 7:**
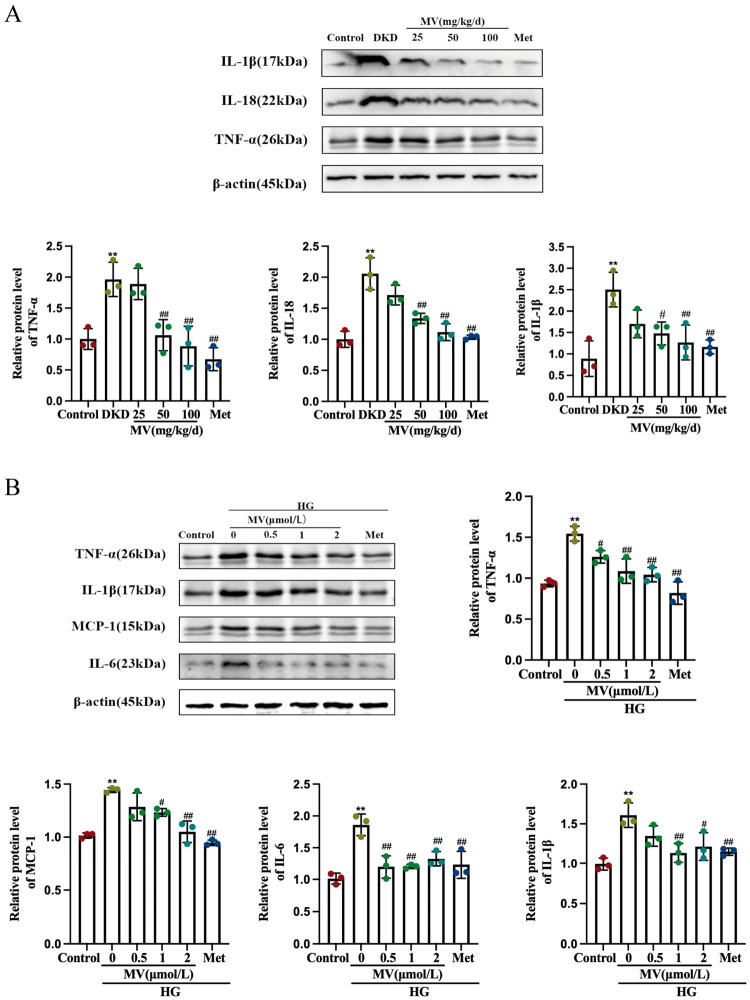
Effect of MV on inflammatory factors. (**A**) Western blot analysis was used to detect the expression of inflammatory proteins TNF-α, IL-1β, and IL-18 in db/db mice. (**B**) Western blot analysis was used to detect the expression of inflammatory proteins TNF-α, IL-1β, MCP-1 and IL-6 in HRMC. ** *p* < 0.01 vs. respective control group; # *p* < 0.05 vs. respective DKD group (DKD mice for panel A, HG-treated HRMC for panel B); ## *p* < 0.01 vs. respective DKD group.

To further evaluate the anti-inflammatory effects of MV, we conducted Western blot experiments to detect the protein expression levels of IL-1β, TNF-α, IL-18, IL-6, and MCP-1 in the kidneys of db/db mice and HG-induced HRMC. Figure 7A,B results showed that in the model group, the protein expression levels of these inflammatory cytokines were significantly increased (*p* < 0.01), while under drug treatment, the expression of these proteins decreased significantly with statistical significance (*p* < 0.01). This demonstrates that MV attenuates inflammatory reactions at both animal and cellular levels.

### 2.6. Effect of MV on TLR4/MyD88/NF-κB Inflammasome Pathways

**Figure 8:**
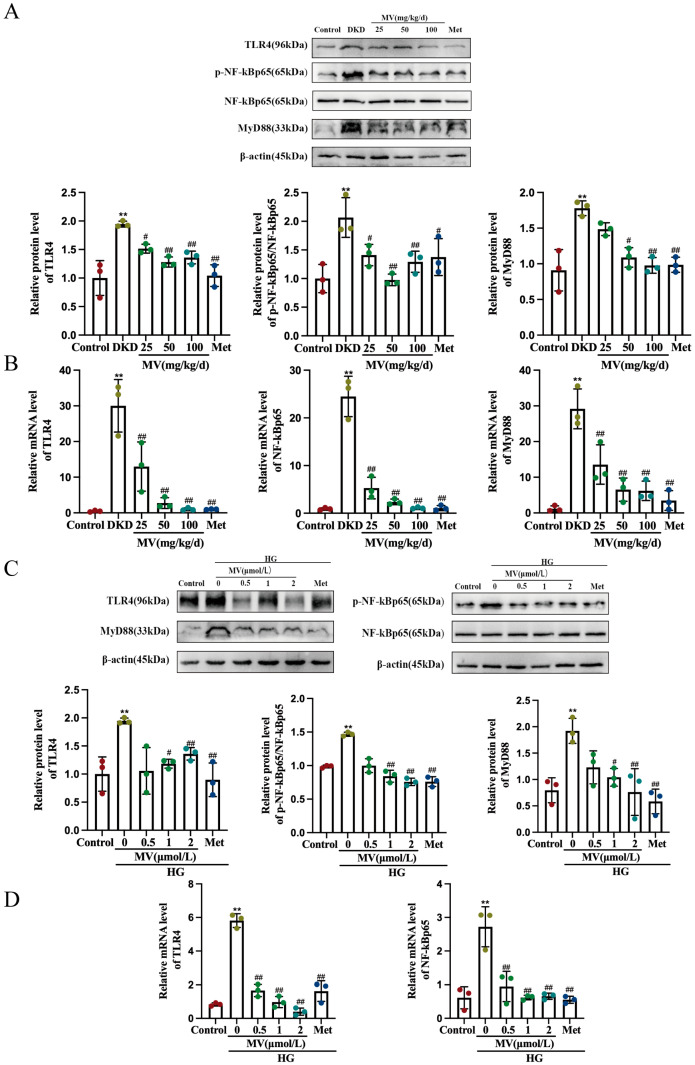
Effect of MV on *TLR4*/*MyD88*/*NF-κB* signal path. (**A**) Western blot analysis was used to detect the protein expression of *TLR4*, *MyD88*, *NF-κB* in db/db mice. (**B**) The mRNA levels of *TLR4*, *MyD88*, and *NF-κB* were measured by qRT-PCR in db/db mice. (**C**) Western blot analysis was used to detect the proteins expression of *TLR4*, *MyD88*, and *NF-κB* in HRMC. (**D**) The mRNA levels of *TLR4* and *NF-κB* were measured by qRT-PCR in HRMC. ** *p* < 0.01 vs. respective control group; # *p* < 0.05 vs. respective DKD group (DKD mice for panel A, HG-treated HRMC for panel B); ## *p* < 0.01 vs. respective DKD group.

To investigate the effect of MV on the *TLR4*/*NF-κB* pathway, Western blotting and RT-qPCR were used to detect the expression levels of *TLR4*, *MyD88*, NF-κBp65, and p-NF-κBp65 proteins and mRNA in mouse renal tissue and HG-induced HRMC. Figure 8A–D results showed that the expression levels of *TLR4*, *MyD88*, and p-NF-κBp65 were significantly increased in the model group (*p* < 0.01), while MV and Met could inhibit this increase (*p* < 0.05, *p* < 0.01). These findings suggest that the effects of MV may be related to the regulation of the *TLR4*/*MyD88*/*NF-κB* pathway.

**Figure 9:**
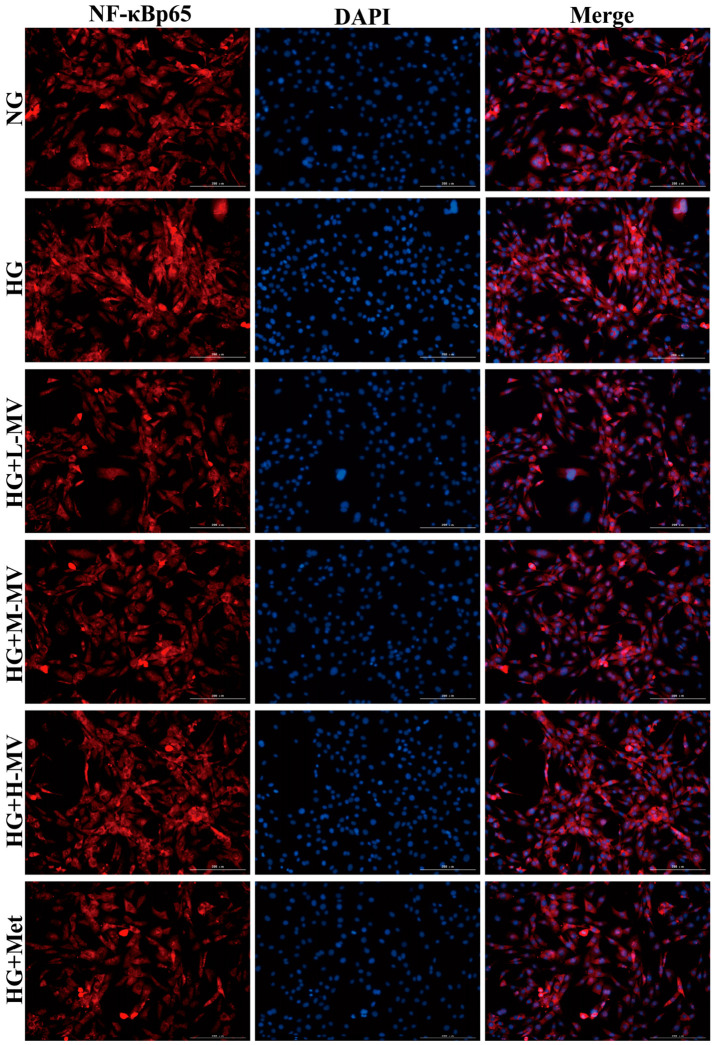
Effect of MV on nuclear NF-κBp65 transport induced by HG in HRMCs. *NF-κB* staining showed red fluorescence and nucleus staining showed blue fluorescence.

The *NF-κB* activation–nuclear transport test was further verified. As shown in Figure 9, the red fluorescence is NF-κBp65 and the blue fluorescence is DAPI. Compared to control group, the red fluorescence in the HG group was enhanced. The results showed that NF-κBp65 transferred into the nucleus in HG environment. After administration of MV or Met, the red fluorescence in the nucleus was significantly weaker than that in the HG group, suggesting that MV could inhibit the nuclear transport of NF-κBp65 in HRMCs. It is further proved that the effect of MV may be related to the downregulation of the *TLR4*/*MyD88*/*NF-κB* signaling pathway.

**Figure 10:**
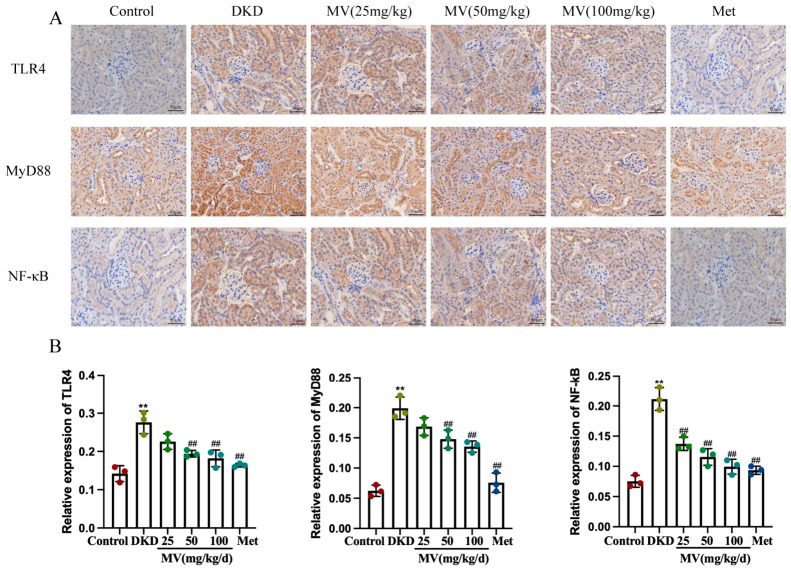
Effect of MV on the levels of *TLR4*, *MyD88*, and *NF-κB* in the kidney of db/db mice. (**A**) Immunohistochemical map of *TLR4*, *MyD88*, and *NF-κB* (400×). (**B**) Immunohistochemical optical density analysis of *TLR4*, *MyD88*, and *NF-κB* proteins in kidney tissue. ** *p* < 0.01 vs. control group; ## *p* < 0.01 vs. DKD group.

In addition, the IHC method was employed to delve into the role of MV in the *TLR4*/*NF-κB* pathway. Figure 10 results showed that compared to the blank group, the expression levels of *TLR4*, *MyD88*, and *NF-κB* were significantly elevated in model group db/db mice (*p* < 0.01). However, after eight weeks of treatment, the expression levels of *TLR4*, *MyD88*, and *NF-κB* in db/db mice were significantly reduced (*p* < 0.05, *p* < 0.01), suggesting that the effects of MV may be associated with the decreased expression of *TLR4*, *MyD88*, and *NF-κB* in mouse kidneys.

## 3. Discussion

Diabetic kidney disease (DKD), as the most common microvascular complication of diabetes mellitus, triggers the excessive release of inflammatory cytokines such as tumor necrosis factor-α, interleukin-1β, and interleukin-6, thereby disrupting renal homeostasis [32], inducing apoptosis and excessive extracellular matrix (ECM) deposition [33], leading to progressive decline in renal function and eventually end-stage renal disease. Therefore, the key pathological features of DKD are uncontrolled chronic renal inflammation, aberrant ECM remodeling, and the impairment of the glomerular filtration barrier. During the progression of diabetic renal injury, high glucose and advanced glycation end-products activate Toll-like receptor 4 (*TLR4*), initiate the *NF-κB* signaling axis, induce the transcription and release of pro-inflammatory cytokines, and thus trigger and accelerate diabetic renal injury [21]. Mogroside V (MV) is a tetracyclic triterpenoid saponin derived from *Siraitia grosvenorii* and possesses hypoglycemic, anti-inflammatory, and antioxidant activities [27,28]. The present study demonstrates that MV ameliorates renal injury in db/db diabetic mice and high-glucose-induced human renal mesangial cell (HRMC) injury.

Animal experiments showed that eight weeks of MV intervention significantly reduced fasting blood glucose (FBG), the area under the curve of oral glucose tolerance test (OGTT AUC), serum creatinine (Scr), blood urea nitrogen (BUN), and 24 h urinary protein levels, and alleviated the renal hypertrophy index in db/db mice (Figure 2). Histopathological examination revealed that MV attenuated glomerular basement membrane (GBM) thickening, mesangial matrix expansion, and collagen deposition (Figure 5A,B). These results are consistent with the report by Suzuki et al. that the *Siraitia grosvenorii* extract reduced urinary protein excretion in diabetic rats [34].

Apoptosis and excessive ECM deposition are critical pathological events in the progression of DKD [35,36]. At the cellular level, high glucose (25 mmol/L) for 48 h significantly inhibited the proliferation of HRMC, and MV (0.5–10 μM) dose-dependently reversed this inhibition, with the most pronounced pro-proliferative effect observed at 2 μM (Figure 3). *Bcl-2*, *Bax*, and cleaved *caspase-3* are core molecules regulating apoptosis; their aberrant expression accelerates renal cell injury. High glucose markedly increased the expression of *Bax* and cleaved *caspase-3* and decreased the *Bcl-2*/*Bax* ratio in HRMC, promoting apoptosis, whereas MV treatment significantly reversed these abnormalities (Figure 4). Collagen type IV (*Col IV*) and matrix metalloproteinase-9 (*MMP-9*) are key indicators of ECM deposition and remodeling; their elevated expression aggravates glomerulosclerosis and tubulointerstitial fibrosis [5,37,38]. In kidney tissues of db/db mice and HG-induced HRMC, the expression of *Col IV* and *MMP-9* was significantly upregulated, indicating excessive ECM accumulation, while MV intervention markedly reduced their expression and improved ECM remodeling imbalance (Figure 5C,D). In this study, MV significantly ameliorated HG-induced HRMC apoptosis, decreased cleaved *caspase-3* expression, increased the *Bcl-2*/*Bax* ratio, and downregulated *MMP-9* and *Col IV* expression, thereby reducing excessive ECM deposition.

In DKD, pro-inflammatory cytokines (e.g., TNF-α, IL-1β, IL-6, MCP-1, IL-18) mainly activate resident renal cells and recruit circulating immune cells into the kidney, jointly driving glomerular filtration barrier disruption and renal interstitial fibrosis. TNF-α upregulates the expression of multiple pro-inflammatory factors, increases glomerular capillary permeability, and accelerates leukocyte infiltration into renal tissue, leading to glomerular hypertrophy and interstitial injury [9,39,40]. IL-6 promotes the local infiltration of macrophages and inflammatory cells in the kidney, further aggravating tubulointerstitial injury and renal fibrosis; its excessive release also amplifies local inflammation and promotes the persistent progression of renal injury, thereby accelerating DKD [10,41]. *TLR4*, an important member of the pattern recognition receptor family, is highly expressed in renal tissue of DKD and, through the activation of the downstream *MyD88*-dependent pathway, promotes *NF-κB* nuclear translocation, subsequently inducing the transcription and release of the above-mentioned pro-inflammatory cytokines [17,21,42,43]. In the present study, the expression of *TLR4*, *MyD88*, and p-NF-κBp65 was significantly increased in kidney tissues of db/db mice and HG-induced HRMC (Figure 8), accompanied by markedly elevated levels of pro-inflammatory cytokines in serum and cell supernatants (Figure 6 and Figure 7). After MV treatment, the expression of *TLR4*/*MyD88*/*NF-κB* pathway-related proteins was significantly downregulated, and the levels of pro-inflammatory cytokines decreased, suggesting that the renoprotective effect of MV may be associated with the inhibition of this pathway. Immunofluorescence staining showed reduced NF-κB p65 nuclear translocation (Figure 9), and immunohistochemical analysis further confirmed that MV reduced the expression of *TLR4*, *MyD88*, and *NF-κB* in renal tissue (Figure 10). These results indicate a consistent trend between the decrease in *TLR4*/*NF-κB* pathway activity and the reduction in pro-inflammatory cytokine levels after MV treatment, further suggesting that the renoprotective effect of MV may be related to downregulating this pathway.

The protective effect of MV against DKD is characterized by multi-target and multi-pathway synergy. Taken together, our findings demonstrate that MV attenuates inflammation by downregulating the *TLR4*/*NF-κB* pathway, exerts anti-fibrotic effects by modulating ECM metabolism, and simultaneously exhibits anti-apoptotic activity. This study validates the regulatory effect of MV on the *TLR4*/*MyD88*/*NF-κB* pathway in mesangial cells and integrates its anti-inflammatory effect with its anti-fibrotic (reducing *MMP-9*, *Col IV*) and anti-apoptotic (regulating *Bcl-2*/*Bax*, *caspase-3*) actions, revealing a network regulatory mechanism underlying the multi-target synergistic protection of MV in DKD. Previous studies have shown that *TLR4* promotes tubular inflammation in DKD [21] and mediates macrophage infiltration [8]; moreover, multiple bioactive compounds derived from medicinal plants, such as resveratrol, curcumin, and paeoniflorin, have been reported to improve DKD via the blockade of the TLR4-mediated inflammatory cascade [17,43,44,45,46]. The present study links the action of MV to this key pathway, providing a novel molecular mechanism clue for the treatment of DKD with MV.

However, certain limitations of this study should be acknowledged. Although we observed synchronous changes in *TLR4*/*NF-κB* pathway-related protein expression and inflammatory cytokine levels after MV intervention, this evidence is correlative. We did not employ specific *TLR4* inhibitors (e.g., TAK-242) or gene knockdown (siRNA) interventions to establish a direct causal relationship. Future studies using *TLR4* inhibitors or gene knockdown experiments will help clarify the direct mechanism of action, thereby providing more substantial scientific evidence for the clinical translation of MV as a functional food or adjunctive therapy.

## 4. Materials and Methods

### 4.1. Drugs and Reagents

Mogroside V (MV, HPLC ≥98%, Lot: PRF10102441) was purchased from Chengdu Purify Technology Development Co., Ltd. (Chengdu, Sichuan, China). Metformin hydrochloride (Met, Cat: 2008058) was purchased from Jingfeng Pharmaceutical Group Co., Ltd. (Beijing, China). D-(+)-Glucose (Cat: G8270) was purchased from Sigma-Aldrich (St. Louis, MO, USA). Human renal mesangial cells (HRMCs) were obtained from Guangzhou Jennio Biotech Co., Ltd. (Guangzhou, Guangdong, China). MEM medium (Cat: C11095500BT), fetal bovine serum (FBS, Cat: 10099141), and penicillin–streptomycin (Cat: 15140122) were purchased from Thermo Fisher Scientific (Waltham, MA, USA). Trypsin-EDTA (Cat: T1300), RIPA buffer (Cat: R0010), PMSF (Cat: P0100), BCA protein concentration determination kit (Cat: PC0020), and MTT (Cat: T1320) were purchased from Beijing Solarbio Science & Technology Co., Ltd. (Beijing, China). ELISA kits for TNF-α (Cat: EK182), IL-1β (Cat: EK101B), IL-6 (Cat: EK106), MCP-1 (Cat: EK187), and IL-18 (Cat: EK118) were purchased from Hangzhou Lianke Biotech Co., Ltd. (Hangzhou, China). The ECL chemiluminescence substrate (Cat: D046) was purchased from Bridgen (Beijing, China). The protein prestain marker (Cat: 26616) and PVDF membrane (Cat: IPVH00010) were purchased from Thermo Fisher Scientific (Waltham, MA, USA). *TLR4* antibody (Cat: ab13556) was purchased from Abcam (Cambridge, UK). *MyD88* antibody (Cat: 4283S), *NF-κB* p65 antibody (Cat: 8242S), p-NF-κB p65 (Ser536) antibody (Cat: 3033S), *Bcl-2* antibody (Cat: 3498S), *Bax* antibody (Cat: 5023S), *MMP-9* antibody (Cat: 3852S), and β-actin antibody (Cat: 3700S) were purchased from Cell Signaling Technology (Beverly, MA, USA). *Col IV* antibody (Cat: 50273S) was purchased from Cell Signaling Technology. Cleaved *caspase-3* antibody (Cat: sc-56053) was purchased from Santa Cruz Biotechnology (Dallas, TX, USA). HRP-conjugated goat anti-rabbit IgG (Cat: 7074S) and HRP-conjugated goat anti-mouse IgG (Cat: 7076S) secondary antibodies were purchased from Cell Signaling Technology. All chemicals and reagents used were of analytical grade.

### 4.2. Cell Culture

HRMC were obtained from Guangzhou Jennio Biotech Co., Ltd. (Guangzhou, Guangdong, China). HRMC were inoculated in MEM containing 10% fetal bovine serum, 100 U ml-1 penicillin, and 100 μg ml-1 streptomycin at 37 °C with 5% CO_2_. The experimental cells were used between the third and 10th generations as pretreated HRMC.

### 4.3. Animals and Treatment

SPF-grade male C57BL/KsJ/db-/- mice (five weeks old) were purchased from GemPharmatech Co., Ltd. (Nanjing, Jiangsu, China), license number: SCXK2018-0008. All experimental animals were maintained in SPF conditions: four mice per cage, at a temperature of 22 ± 2 °C and a humidity level of 50 ± 10%, under a 12 h light–dark cycle. The animals had ad libitum access to food and water throughout the experiment. The animal study protocol was approved by the Institutional Animal Care and Use Committee (IACUC) of Guilin Medical University (protocol code GLMC-IACUC-2024 1046, date of approval: 20 May 2024).

The db/db mice were randomly divided into five groups, with six mice in each group, and 6 db/m mice in the control group. After one week of adaptive feeding, different concentrations of MV or Met were administered by oral gavage daily: positive group (101.4 mg/kg/d metformin), MV low-dose group (25 mg/kg/d), MV medium-dose group (50 mg/kg/d), MV high-dose group (100 mg/kg/d). The same dose of 0.9% sodium chloride solution was administered to the blank and model groups for eight weeks. The fasting blood glucose (FBG) and glucose tolerance of mice were measured by glucose meter (Accu-Chek Active Blood Glucose Meter, Roche, Cork, Ireland), and the weight of the mice was recorded every week.

### 4.4. Analysis of Cell Viability

In order to establish the model of hyperglycemia injury, HRMC was inoculated in 96-well plate with 4 × 10^4^ cell/mL, cultured for 12 h, treated with different concentrations of HG for 12 h, 24 h, 36 h and 48 h, and treated with different concentrations of MV or Met according to the best modeling conditions. The optical density was measured at 490 nm wavelength by MTT colorimetric assay (Agilent Company, Santa Clara, CA, USA), and the effect of MV or Met on HRMC damage induced by high glucose was observed.

### 4.5. Flow Cytometry Analysis

HRMCs in logarithmic growth phase were made into cell suspension containing 8 × 10^4^ cell/mL, which were evenly inoculated in 6-well cell culture plate. After the cells were attached, the cells were intervened in groups for 48 h. The cells of each group were collected in the corresponding centrifuge tube, washed twice by PBS and re-suspended by 1× BindingBuffer. Then, add 10 μL FITC Annexin V, mix gently and incubate 20 min at 25 °C. Then, 10 μL PI was added and incubated for 10 min at 25 °C without light. The cells were filtered into 5 ml flow cell tube by professional filter, and the apoptosis rate was calculated by computer detection within 1 h.

### 4.6. Detection of Biochemical Indexes and Inflammatory Factors

The determination of Scr and BUN in serum and 24 h UALB in urine of mice was conducted by automatic biochemical analyzer. In addition, the concentrations of TNF-α, IL-1β, MCP-1, IL-6, and IL-18 were detected in mouse serum using Elisa assay kits. Furthermore, the concentrations of TNF-α, IL-1β, MCP-1, and IL-18 were also determined in cells.

### 4.7. HE and Masson Staining

For the HE staining, mouse kidney tissues from each group were embedded, sectioned, and deparaffinized. The sections were then immersed in a solution of hematoxylin and eosin for differentiation, followed by rinsing with 1% hydrochloric acid, returning to blue with 1% ammonium hydroxide. After water washing, the sections were stained with eosin and immersed in xylene. The sealed sections were observed under a microscope at 400× magnification and photographed.

As for the Masson staining, mouse kidney tissues from each group were embedded, sectioned, and deparaffinized. The sections were then immersed in a solution of hematoxylin and stained with acid alcohol for differentiation, followed by rinsing with bluing reagent and immersion in light green. Further washing with weak acid working solution was applied before immersing the sections into phosphomolybdic acid and weak acid working solution, followed by immersion into aniline blue and weak acid working solution. After dehydration, the sections were immersed in xylene and fixed with neutral mounting medium. The sealed sections were observed under a microscope at 400× magnification and photographed.

### 4.8. Immunological Histological Chemistry

After embedding the mouse kidney tissues from each group, they were sectioned and deparaffinized. Antigen retrieval was performed by heating the sections in a citrate buffer solution. After cooling, the sections were washed with PBS and then incubated with 3% H_2_O_2_ for 10 min. Following another wash with PBS, the appropriate dilution of primary antibody was added and incubated for 1 h. Afterwards, an enhancement solution was added and incubated for 20 min before being washed with PBS. The secondary antibody was then added and incubated for another 20 min. DAB chromogenic substrate was applied to visualize the staining, and the sections were differentiated in 1% hydrochloric acid and returned to blue. The sections were then dehydrated and immersed in xylene, followed by sealing of the sections onto slides. The sealed sections were observed under a microscope at 400× magnification and photographed. Image J software was used to process the images.

### 4.9. Real-Time Fluorescence PCR

Extract total RNA from cells and kidney using Trizol. Measure the purity and concentration of RNA with a nucleic acid analyzer. According to the instructions of the reverse transcription kit and real-time fluorescent PCR kit, prepare the reaction system. Amplify the samples in a PCR instrument by placing the PCR plate into the machine. Use Actin as an internal reference, and calculate the relative mRNA expression levels using the 2^−ΔΔCt^ method.

The primer sequences for the animal experiment are listed below: *TLR4*, 5′-ATCCAACACTAAGGAGGTAT-3′ (forward) and 5′-GGTCAAGGAACAGAAGCA-3′ (reverse); *MyD88*, 5′-GATGCCTTTATCTGCTACTG-3′ (forward) and 5′-GCGACACCTTTTCTCAAT-3′ (reverse); *NF-κB*, 5′-CTACGGTGGGATTACATT-3′ (forward) and 5′-TCTCCTCGTCATCACTCTT-3′ (reverse); *MMP-9*, 5′-CGGCAACGGAGAAGGCAAAC-3′ (forward) and 5′-GTGGCGCACCAGCGGTAA-3′ (reverse); *β-actin*, 5′-TCATCACTATTGGCAACGAGC-3′ (forward) and 5′-AACAGTCCGCCTAGAAGCAC-3′ (reverse).

The primer sequence for the cell experiment is listed below: *TLR4*, 5′- TACAAAATCCCCGACAACCTCC-3′ (forward) and 5′- GCTGCCTAAATGCCTCAGGG-3′ (reverse); *NF-κB*, 5′-AGAGCAGCGTGGGGACTA-3′ (forward) and 5′- ATGGGATGAGAAAGGACAGG-3′ (reverse); *β-actin*, 5′-CGCGAGAA-GATGACCCAGAT-3′ (forward), and 5′-GGGCATACCCCTCGTAGATG-3′ (reverse).

### 4.10. Western Blotting Analysis

Load the protein sample into the wells for electrophoresis, transfer, and blocking on PVDF membrane. Incubate the PVDF membrane with diluted primary antibody (1:1500) overnight at 4 °C. Wash the PVDF membrane three times with TBST for 10 min each time. Incubate with secondary antibody (1:10,000) for 1 h and wash with TBST three more times for 10 min each. Finally, drop ECL luminescent agent onto the strip and obtain the gel image using a chemiluminescence imaging system.

### 4.11. NF-κB Activation—Nuclear Transport Detection

The method of cell treatment was the same as the determination of glucose consumption. After 48 h of intervention, the supernatant of cell culture was discarded and washed with PBS. Then, after the activation of *NF-κB*, the main subunit p65 of *NF-κB* was transported into the nucleus. It was observed under inverted fluorescence microscope.

### 4.12. Statistical Analysis

The statistical analysis of the data was performed using SPSS Statistics 25 software, and GraphPad Prism 8.0.1 software was used to generate graphs. The differences between groups were compared using either a T-test or one-way ANOVA, and the results were expressed as mean ± SEM or SD for the statistical data. A significant difference was defined as *p* < 0.05.

## 5. Conclusion

In this study, Mogroside V exhibited promising protective effects against diabetic kidney disease in db/db mice and HG-induced HRMC injury models. Its mechanisms are related to the regulation of the *TLR4*/*MyD88*/*NF-κB* signaling pathway, thereby attenuating inflammatory responses, reducing extracellular matrix accumulation, and suppressing renal cell apoptosis. These findings not only elucidate the multi-target synergistic protective effects of MV against DKD but also provide a scientific basis for the further development of MV as a functional food or adjunctive therapy. Moreover, this study yields valuable information for the in-depth development and utilization of *Siraitia grosvenorii* as a medicinal and edible plant resource.

## Figures and Tables

**Table 1:** Changes in body weight of db/db mice during administration ($\bar{x}$ ± s, *n* = 6, g).

Group	Week 0	Week 2	Week 4	Week 6	Week 8
Control	20.62 ± 0.57	21.04 ± 0.46	21.39 ± 0.72	22.36 ± 1.11	22.72 ± 1.24
DKD	32.70 ± 1.23	38.22 ± 2.61	43.96 ± 2.42	42.49 ± 3.10	39.58 ± 3.70
MV25 mg/kg/d	33.35 ± 1.95	39.68 ± 3.36	44.93 ± 3.47	48.43 ± 3.54 ^##^	51.49 ± 3.33 ^##^
MV50 mg/kg/d	31.95 ± 1.12	39.48 ± 2.20	44.72 ± 3.55	48.18 ± 4.12 ^##^	50.64 ± 3.83 ^##^
MV100 mg/kg/d	32.47 ± 1.95	40.62 ± 1.33	45.08 ± 1.75	48.17 ± 2.51 ^##^	50.98 ± 3.14 ^##^
Met	31.69 ± 0.83	38.36 ± 1.74	43.08 ± 1.82	47.23 ± 1.45 ^##^	49.43 ± 2.74 ^##^

Note: ^##^ *p* < 0.01 vs. DKD group.

## Data Availability

Data will be made available on request.

## Abbreviations

The following abbreviations are used in this manuscript:

BUN	Blood urea nitrogen
*Col IV*	Collagen type IV
DKD	Diabetic kidney disease
ECM	Extracellular matrix
ELISA	Enzyme-linked immunosorbent assay
FBG	Fasting blood glucose
GBM	Glomerular basement membrane
HE	Hematoxylin and eosin
HG	High glucose
HRMC	Human renal mesangial cells
IHC	Immunohistochemistry
IL-1β	Interleukin-1β
IL-6	Interleukin-6
IL-18	Interleukin-18
*MCP-1*	Monocyte chemoattractant protein-1
Met	Metformin
*MMP-9*	Matrix metalloproteinase-9
MTT	3-(4,5-Dimethylthiazol-2-yl)-2,5-diphenyltetrazolium bromide
MV	Mogroside V
*MyD88*	Myeloid differentiation factor 88
*NF-κB*	Nuclear factor kappa-B
OGTT	Oral glucose tolerance test
PVDF	Polyvinylidene fluoride
RT-qPCR	Real-time quantitative polymerase chain reaction
Scr	Serum creatinine
SEM	Standard error of mean
*TLR4*	Toll-like receptor 4
TNF-α	Tumor necrosis factor-α
24 h UALB	24 h urinary albumin

## References

[1] Selby N.M., Taal M.W. (2020). An updated overview of diabetic nephropathy: Diagnosis, prognosis, treatment goals and latest guidelines. Diabetes Obes. Metab..

[2] American Diabetes Association Professional Practice Committee (2025). 2. Diagnosis and Classification of Diabetes: Standards of Care in Diabetes-2025. Diabetes Care.

[3] Tofte N., Lindhardt M., Adamova K., Bakker S.J.L., Beige J., Beulens J.W.J., Birkenfeld A.L., Currie G., Delles C., Dimos I. (2020). Early detection of diabetic kidney disease by urinary proteomics and subsequent intervention with spironolactone to delay progression (PRIORITY): A prospective observational study and embedded randomised placebo-controlled trial. Lancet Diabetes Endocrinol..

[4] Shetty S., Suvarna R., Awasthi A., Bhojaraja M.V., Pappachan J.M. (2025). Emerging Biomarkers and Innovative Therapeutic Strategies in Diabetic Kidney Disease: A Pathway to Precision Medicine. Diagnostics.

[5] Garcia-Fernandez N., Jacobs-Cachá C., Mora-Gutiérrez J.M., Vergara A., Orbe J., Soler M.J. (2020). Matrix Metalloproteinases in Diabetic Kidney Disease. J. Clin. Med..

[6] Ismail-Beigi F., Craven T., Banerji M.A., Basile J., Calles J., Cohen R.M., Cuddihy R., Cushman W.C., Genuth S., Grimm R.H. (2010). Effect of intensive treatment of hyperglycaemia on microvascular outcomes in type 2 diabetes: An analysis of the ACCORD randomised trial. Lancet.

[7] Husain-Syed F., Yuecel G., Daschner C., Jochims J., Yazdani B. (2026). Therapeutic Advances in Diabetic Kidney Disease: 30 Years of Evidence and the Rise of the “Fantastic Four” in Nephrology. Cardiorenal Med..

[8] Satirapoj B., Adler S.G. (2014). Comprehensive approach to diabetic nephropathy. Kidney Res. Clin. Pract..

[9] Chen Y., Li H., Dai Q., Tan Z., Wu H., Xu Z., Wang G., Fang Y., Luo J., Yu C. (2025). The pathogenesis of diabetic kidney disease and the therapeutic potential of bioactive substances. Front. Pharmacol..

[10] Jin Q., Liu T., Qiao Y., Liu D., Yang L., Mao H., Ma F., Wang Y., Peng L., Zhan Y. (2023). Oxidative stress and inflammation in diabetic nephropathy: Role of polyphenols. Front. Immunol..

[11] Thomas H.Y., Ford Versypt A.N. (2022). Pathophysiology of mesangial expansion in diabetic nephropathy: Mesangial structure, glomerular biomechanics, and biochemical signaling and regulation. J. Biol. Eng..

[12] Feng L., Feng Y.Y., Ren Q., Fu P., Ma L. (2025). Mesangial Cells in Diabetic Kidney Disease: From Mechanisms to Therapeutic Implications. Int. J. Biol. Sci..

[13] Liu X., Luo F., Pan K., Wu W., Chen H. (2007). High glucose upregulates connective tissue growth factor expression in human vascular smooth muscle cells. BMC Cell Biol..

[14] Maki T., Maeno S., Maeda Y., Yamato M., Sonoda N., Ogawa Y., Wakisaka M., Inoguchi T. (2019). Amelioration of diabetic nephropathy by SGLT2 inhibitors independent of its glucose-lowering effect: A possible role of SGLT2 in mesangial cells. Sci. Rep..

[15] Kim D.I., Park M.J., Heo Y.R., Park S.H. (2015). Metformin ameliorates lipotoxicity-induced mesangial cell apoptosis partly via upregulation of glucagon like peptide-1 receptor (GLP-1R). Arch. Biochem. Biophys..

[16] Zhang Q.Y., Xu S.J., Qian J.C., Yang L.B., Chen P.Q., Wang Y., Hu X., Zhang Y.L., Luo W., Liang G. (2022). Pharmacological inhibition of *MyD88* suppresses inflammation in tubular epithelial cells and prevents diabetic nephropathy in experimental mice. Acta Pharmacol. Sin..

[17] Wu J., Li K., Zhou M., Gao H., Wang W., Xiao W. (2024). Natural compounds improve diabetic nephropathy by regulating the *TLR4* signaling pathway. J. Pharm. Anal..

[18] Shelke V., Dagar N., Gaikwad A.B. (2025). Renoprotective Effects of Phloretin and TUDCA via Simultaneous Inhibition of *TLR4*/*MyD88*/*NF-κB* and BiP/PERK/CHOP Pathways in AKI Under Diabetic Condition. Appl. Biochem. Biotechnol..

[19] Nikolaidis C.G., Gyriki D., Stavropoulou E., Karlafti E., Didangelos T., Tsigalou C., Thanopoulou A. (2025). Targeting the *TLR4* axis with microbiota-oriented interventions and innovations in diabetes therapy: A narrative review. Front. Immunol..

[20] Yu Y., Zhang Y., Tang Y., Ma L., Wei C., Bai X. (2026). Advanced Glycation End-Products Contribute to Delayed Diabetic Corneal Epithelial Wound Healing via the *TLR4* Signaling. Investig. Ophthalmol. Vis. Sci..

[21] Lin M., Yiu W.H., Wu H.J., Chan L.Y., Leung J.C., Au W.S., Chan K.W., Lai K.N., Tang S.C. (2012). Toll-like receptor 4 promotes tubular inflammation in diabetic nephropathy. J. Am. Soc. Nephrol..

[22] Verzola D., Cappuccino L., D’Amato E., Villaggio B., Gianiorio F., Mij M., Simonato A., Viazzi F., Salvidio G., Garibotto G. (2014). Enhanced glomerular Toll-like receptor 4 expression and signaling in patients with type 2 diabetic nephropathy and microalbuminuria. Kidney Int..

[23] Lin M., Yiu W.H., Li R.X., Wu H.J., Wong D.W., Chan L.Y., Leung J.C., Lai K.N., Tang S.C. (2013). The *TLR4* antagonist CRX-526 protects against advanced diabetic nephropathy. Kidney Int..

[24] Zhang Y.F., Ma Y.X., Wei S.J., Yang B., Ji Y.H., Qi Z.X., Shi X.Y., Zhang L.L., Fan X.Z., Yang X.J. (2025). A Novel *TLR4* Inhibitor DB03476 Rescued Renal Inflammation in Acute Kidney Injury Model. Int. J. Mol. Sci..

[25] Han M., Liu H., Liu G., Li X., Zhou L., Liu Y., Dou T., Yang S., Tang W., Wang Y. (2024). Mogroside V alleviates inflammation response by modulating miR-21-5P/SPRY1 axis. Food Funct..

[26] Gong X., Chen N., Ren K., Jia J., Wei K., Zhang L., Lv Y., Wang J., Li M. (2019). The Fruits of *Siraitia grosvenorii*: A Review of a Chinese Food-Medicine. Front. Pharmacol..

[27] Huang H., Peng Z., Zhan S., Li W., Liu D., Huang S., Zhu Y., Wang W. (2024). A comprehensive review of *Siraitia grosvenorii* (Swingle) C. Jeffrey: Chemical composition, pharmacology, toxicology, status of resources development, and applications. Front. Pharmacol..

[28] Chen N., Cao W., Yuan Y., Wang Y., Zhang X., Chen Y., Yiasmin M.N., Tristanto N.A., Hua X. (2024). Recent advancements in mogrosides: A review on biological activities, synthetic biology, and applications in the food industry. Food Chem..

[29] Shi D., Zheng M., Wang Y., Liu C., Chen S. (2014). Protective effects and mechanisms of mogroside V on LPS-induced acute lung injury in mice. Pharm. Biol..

[30] Dou T., Wang J., Liu Y., Jia J., Zhou L., Liu G., Li X., Han M., Lin J., Huang F. (2022). A Combined Transcriptomic and Proteomic Approach to Reveal the Effect of Mogroside V on OVA-Induced Pulmonary Inflammation in Mice. Front. Immunol..

[31] Tao L., Yang J., Cao F., Xie H., Zhang M., Gong Y., Zhang C. (2017). Mogroside IIIE, a Novel Anti-Fibrotic Compound, Reduces Pulmonary Fibrosis through Toll-Like Receptor 4 Pathways. J. Pharmacol. Exp. Ther..

[32] Liu P., Zhang Z., Li Y. (2021). Relevance of the Pyroptosis-Related Inflammasome Pathway in the Pathogenesis of Diabetic Kidney Disease. Front. Immunol..

[33] Alicic R.Z., Rooney M.T., Tuttle K.R. (2017). Diabetic Kidney Disease: Challenges, Progress, and Possibilities. Clin. J. Am. Soc. Nephrol..

[34] Suzuki Y.A., Tomoda M., Murata Y., Inui H., Sugiura M., Nakano Y. (2007). Antidiabetic effect of long-term supplementation with *Siraitia grosvenori* on the spontaneously diabetic Goto-Kakizaki rat. Br. J. Nutr..

[35] Liu X., Zhang C., Fu Y., Xie L., Kong Y., Yang X. (2025). Inflammation, Apoptosis, and Fibrosis in Diabetic Nephropathy: Molecular Crosstalk in Proximal Tubular Epithelial Cells and Therapeutic Implications. Curr. Issues Mol. Biol..

[36] Toth-Manikowski S., Atta M.G. (2015). Diabetic Kidney Disease: Pathophysiology and Therapeutic Targets. J. Diabetes Res..

[37] Xu X., Xiao L., Xiao P., Yang S., Chen G., Liu F., Kanwar Y.S., Sun L. (2014). A glimpse of matrix metalloproteinases in diabetic nephropathy. Curr. Med. Chem..

[38] van der Zijl N.J., Hanemaaijer R., Tushuizen M.E., Schindhelm R.K., Boerop J., Rustemeijer C., Bilo H.J., Verheijen J.H., Diamant M. (2010). Urinary matrix metalloproteinase-8 and -9 activities in type 2 diabetic subjects: A marker of incipient diabetic nephropathy?. Clin. Biochem..

[39] Hou G., Dong Y., Jiang Y., Zhao W., Zhou L., Cao S., Li W. (2025). Immune inflammation and metabolic interactions in the pathogenesis of diabetic nephropathy. Front. Endocrinol..

[40] Ansari Z., Chaurasia A., Neha, Sharma N., Bachheti R.K., Gupta P.C. (2025). Exploring inflammatory and fibrotic mechanisms driving diabetic nephropathy progression. Cytokine Growth Factor. Rev..

[41] Chen I.C., Wang S.C., Chen Y.T., Tseng H.H., Liu P.L., Lin T.C., Wu H.E., Chen Y.R., Tseng Y.H., Hsu J.H. (2021). Corylin Ameliorates LPS-Induced Acute Lung Injury via Suppressing the MAPKs and IL-6/STAT3 Signaling Pathways. Pharmaceuticals.

[42] Kaur H., Chien A., Jialal I. (2012). Hyperglycemia induces Toll like receptor 4 expression and activity in mouse mesangial cells: Relevance to diabetic nephropathy. Am. J. Physiol. Ren. Physiol..

[43] Shao Y.X., Gong Q., Qi X.M., Wang K., Wu Y.G. (2019). Paeoniflorin Ameliorates Macrophage Infiltration and Activation by Inhibiting the *TLR4* Signaling Pathway in Diabetic Nephropathy. Front. Pharmacol..

[44] Sun L.N., Yang Z.Y., Lv S.S., Liu X.C., Guan G.J., Liu G. (2014). Curcumin prevents diabetic nephropathy against inflammatory response via reversing caveolin-1 Tyr14 phosphorylation influenced *TLR4* activation. Int. Immunopharmacol..

[45] Sun F., Wang X.H., Fang Z., Wang W., Wang D., Teng J. (2024). Mechanisms of resveratrol in alleviating diabetic nephropathy: Focus on tumor necrosis factor receptor-related factor expression and toll-like reeptor 4/nuclear factor-kappaB signaling pathway. J. Physiol. Pharmacol..

[46] Yang J., Dong H., Wang Y., Jiang Y., Zhang W., Lu Y., Chen Y., Chen L. (2020). Cordyceps cicadae polysaccharides ameliorated renal interstitial fibrosis in diabetic nephropathy rats by repressing inflammation and modulating gut microbiota dysbiosis. Int. J. Biol. Macromol..

